# An integrated framework to identify wildlife populations under threat from climate change

**DOI:** 10.1111/1755-0998.12694

**Published:** 2017-07-25

**Authors:** Orly Razgour, John B. Taggart, Stephanie Manel, Javier Juste, Carlos Ibáñez, Hugo Rebelo, Antton Alberdi, Gareth Jones, Kirsty Park

**Affiliations:** ^1^ Biological Sciences University of Southampton Southampton UK; ^2^ School of Biological Sciences University of Bristol Bristol UK; ^3^ Biological & Environmental Sciences University of Stirling Stirling UK; ^4^ Institute of Aquaculture University of Stirling Stirling UK; ^5^ EPHE PSL Research University CNRS UM SupAgro IND INRA UMR 5175 CEFE Montpellier France; ^6^ Estacion Biológica de Doñana (CSIC) Seville Spain; ^7^ Centro de Investigação em Biodiversidade e Recursos Genéticos da Universidade do Porto (CIBIO/UP) Vairão Portugal; ^8^ Natural History Museum of Denmark University of Copenhagen Copenhagen K Denmark

**Keywords:** bats, conservation genomics, genotype–environment associations, global change, landscape genetics, range shifts

## Abstract

Climate change is a major threat to global biodiversity that will produce a range of new selection pressures. Understanding species responses to climate change requires an interdisciplinary perspective, combining ecological, molecular and environmental approaches. We propose an applied integrated framework to identify populations under threat from climate change based on their extent of exposure, inherent sensitivity due to adaptive and neutral genetic variation and range shift potential. We consider intraspecific vulnerability and population‐level responses, an important but often neglected conservation research priority. We demonstrate how this framework can be applied to vertebrates with limited dispersal abilities using empirical data for the bat *Plecotus austriacus*. We use ecological niche modelling and environmental dissimilarity analysis to locate areas at high risk of exposure to future changes. Combining outlier tests with genotype–environment association analysis, we identify potential climate‐adaptive SNPs in our genomic data set and differences in the frequency of adaptive and neutral variation between populations. We assess landscape connectivity and show that changing environmental suitability may limit the future movement of individuals, thus affecting both the ability of populations to shift their distribution to climatically suitable areas and the probability of evolutionary rescue through the spread of adaptive genetic variation among populations. Therefore, a better understanding of movement ecology and landscape connectivity is needed for predicting population persistence under climate change. Our study highlights the importance of incorporating genomic data to determine sensitivity, adaptive potential and range shift potential, instead of relying solely on exposure to guide species vulnerability assessments and conservation planning.

## 1 INTRODUCTION

Climate change is a major threat to global biodiversity (IPCC, 2013). Increased periods of drought, thermal stress and extreme climatic events are likely to produce a range of new selection pressures (Hoffmann & Sgrò, 2011). The ability of populations to respond to these changes depends on the rate and magnitude of climate change and individual adaptive capacity based on physiological sensitivity to change, phenotypic plasticity, genetic diversity and dispersal ability (Dawson, Jackson, House, Prentice, & Mace, 2011). Many species are already affected by climate change and, as a result, have displayed a variety of responses, including shifting their ranges and changes to phenotypes, genotypes, growth, phenology and ecological relationships (Peñuelas et al., 2013). Hence, understanding how biodiversity responds to climate change requires an interdisciplinary perspective, combining ecological, molecular and environmental approaches, and an integrated assessment of exposure to changing climatic conditions, adaptive potential and movement ability. Yet while exposure is commonly used to assess species vulnerability to climate change, the other aspects of vulnerability, sensitivity and adaptive potential have been largely neglected, thus precluding accurate estimations of species‐specific vulnerability (Butt et al., 2016).

Ecological niche models (ENMs), also known as species distribution models, offer an effective tool for forecasting how climate change may alter future species distributions and patterns of diversity (Elith, Kearney, & Phillips, 2010). ENMs have been used extensively to identify species vulnerable to future changes (Pacifici et al., 2015) and predict global patterns of extinction risk (Urban, 2015). Their popularity is attributed to the availability of fine‐scale climate change scenarios, the relative simplicity of the modelling procedures and the lack of detailed physiological and life history data necessary for parameterizing more complex mechanistic or demographic models (Guisan & Thuiller, 2005; Thuiller et al., 2013). However, predictive modelling studies have been criticized for being oversimplistic because they rarely address evolutionary processes (Thuiller et al., 2013) or integrate genetic data to support and validate predictions (Gotelli & Stanton‐Geddes, 2015).

The study of local adaptations to current climatic gradients can contribute to understanding the ability of populations to persist or adapt to rapid environmental change (Fournier‐Level et al., 2011). Intraspecific variation in climatic tolerance will result in different responses to climate change below the species level, and therefore, geographic areas that are most sensitive can be identified through mapping spatial patterns of local adaptations (Fitzpatrick & Keller, 2015). Recent technological advances and theoretical developments enable investigation of the genetic basis of adaptations and mechanisms of adaptive responses in wild populations (Andrews, Good, Miller, Luikart, & Hohenlohe, 2016; Orsini, Andrew, & Eizaguirre, 2013). Studies researching patterns of genome variation demonstrated how adaptations to climatic conditions can shape the spatial distribution of variation in plants (*Arabidopsis thaliana*, Fournier‐Level et al., 2011) and humans (Hancock et al., 2011). However, most research to date has focused mainly on model organisms and on genes thought to be involved in adaptations to current environmental conditions, rather than predicting responses to future conditions (Manel & Holderegger, 2013).

While some populations can persist through available genetic variation or their adaptive capacity, the persistence of many individuals depends on their ability to track suitable conditions in space through dispersal or by shifting to different habitats (Bellard, Bertelsmeier, Leadley, Thuiller, & Courchamp, 2012). Understanding dispersal is important for predicting species responses to environmental change because it determines both the rate of distributional shifts and the rate of evolutionary adaptation to changing conditions through the spread of adaptive alleles among populations (Travis et al., 2013). Landscape genetics, the study of the effects of environmental heterogeneity on the spatial distribution of genetic variation (Manel, Schwartz, Luikart, & Taberlet, 2003), can help identify barriers to dispersal that are likely to limit species ability to respond to climate change through tracking changes to their environmental niche (Scoble & Lowe, 2010). A further, yet unexplored, application is to infer the effect of landscape connectivity on the probability of evolutionary adaptation through the spatial spread of adaptive variation between populations. Despite its potential as a predictive tool, thus far landscape genetics has been primarily applied in a descriptive manner (Manel & Holderegger, 2013).

**Figure 1:**
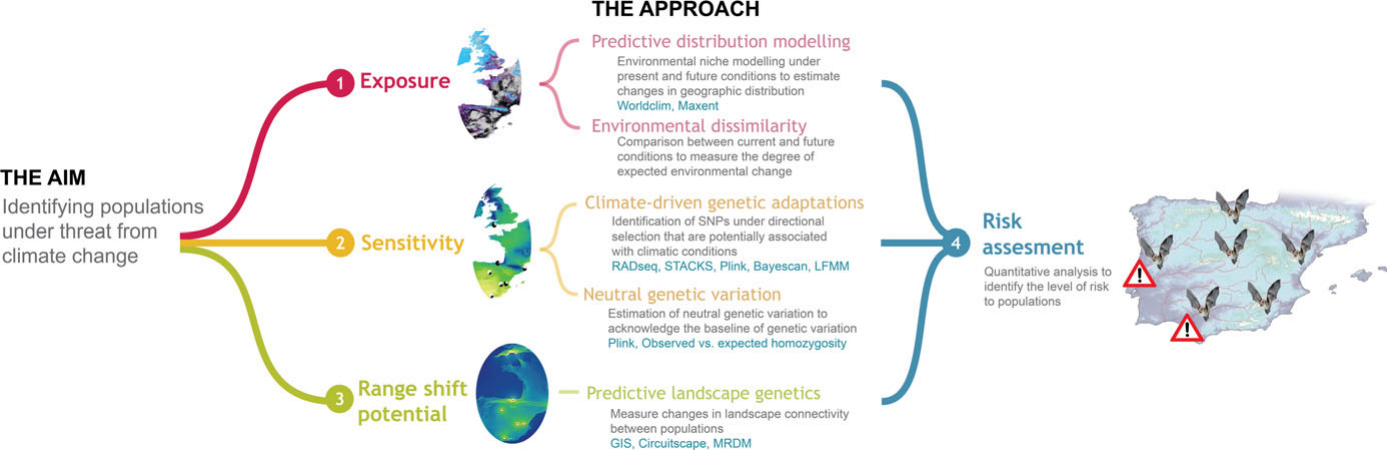
The integrated framework to identify populations under threat from future climate change, including the approaches and methods used to assess the different framework components

We propose an applied framework that integrates ecological, molecular and environmental approaches to identify populations under threat from global climate change. Unlike previous climate change vulnerability assessments (e.g., Pacifici et al., 2015; Pearson et al., 2014), we consider the intraspecific level because populations will go extinct long before species, and it is populations, not species, that are the focus of conservation management. Our framework aims to address the lack of emphasis on sensitivity and adaptive capacity in vulnerability assessments used to inform conservation planning under climate change (Butt et al., 2016). We assess exposure to changing climatic conditions using predictive ENMs and spatial environmental data, sensitivity to climate change using genomic data to identify climate‐driven genetic adaptations, and range shift potential using a predictive landscape genetics approach (Figure 1). This framework is aimed at organisms that are unlikely to genetically adapt fast enough through the spread of novel mutations in the population to keep pace with future changes due to their relatively long lifespans, long generation times and small population sizes (i.e., most vertebrates; Hoffmann & Sgrò, 2011). Therefore, instead of emphasizing general adaptive capacity, like previous conceptual frameworks have done (e.g., Dawson et al., 2011), we focus on the ability to track future climatic suitability (range shift potential) and evolutionary adaptation through the spread of adaptive genetic variation among populations.

We apply our integrated framework to the grey long‐eared bat, *Plecotus austriacus*, a European bat species with relatively limited dispersal ability that is of conservation concern at the northern parts of its range (Van der Meij et al., 2015). We selected this species because its geographic distribution is limited by climate and its current patterns of genetic variation were shaped by past climatic changes (Razgour et al., 2013). Bats possess a number of traits that make them vulnerable to climate change, including low reproductive output, ecological specialization and high trophic positions (Jones & Rebelo, 2013). High surface‐to‐volume ratios due to large membranous, noninsulated wings, mean that evaporative water loss is higher in bats than in other small mammals (Webb, Speakman, & Racey, 1995). As a result, bats may require specific physiological adaptations to cope with increased temperatures and aridity (Muñoz‐Garcia et al., 2016). We aim to identify *P. austriacus* populations vulnerable to future climate change based on their extent of exposure to changing climatic conditions, sensitivity due to adaptive and neutral genetic variation and range shift potential. We use this case study to demonstrate how our integrated framework can inform conservation management under global environmental change.

## 2 MATERIALS AND METHODS

### 2.1 Sampling design

**Figure 2:**
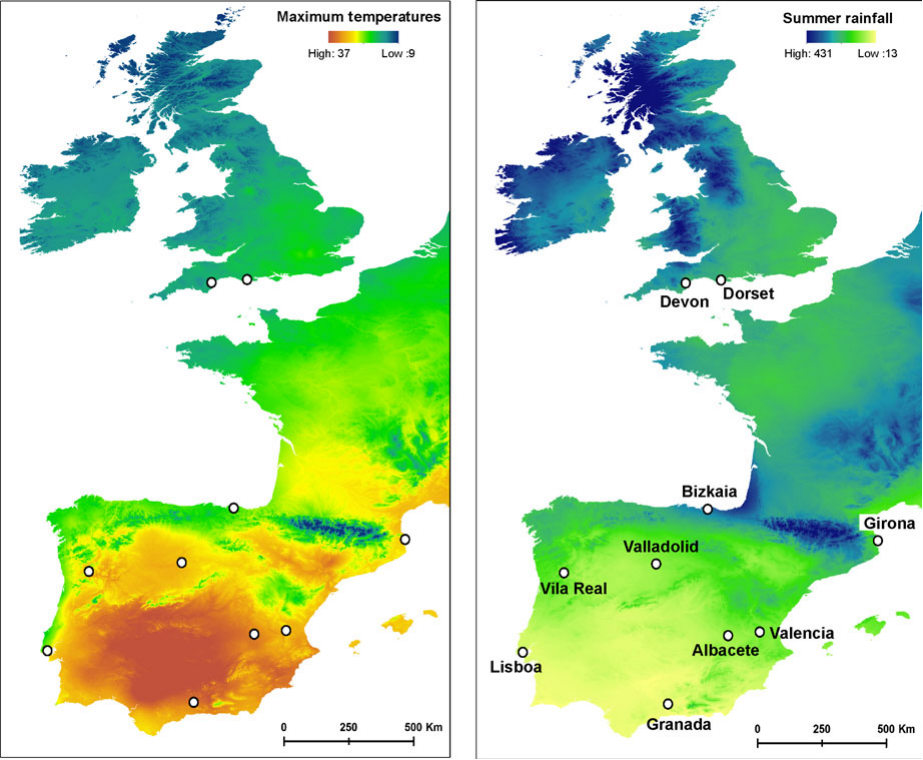
*Plecotus austriacus* populations included in the study presented over maps of maximum temperatures and summer rainfall (http://www.worldclim.org)

We collected nonlethal tissue samples (wing biopsies) from *Plecotus austriacus* bats, sampled between 2009 and 2013 from across the Iberian Peninsula (Iberia) and the south of England. These areas represent the species’ southern and northern range limits, as well as the centre and margin of the species’ ecological niche, respectively. We included 10 populations (eight from Iberia, two from England) that had at least eight individuals with sufficiently high DNA quantity and quality (*N* = 94). These populations represent different geographic areas and combinations of climatic conditions (Figure 2; Table 1). All populations were located more than 90 km apart, exceeding the maximum recorded dispersal distance in this species (62 km; Riede, 2001).

**Table 1:** *Plecotus austriacus* populations included in the final genomic data set with location, region, geographic area within the region, GPS coordinates (WGS1984), number of individuals and average population SNP data set coverage

Population	Year	Region	Area	Latitude	Longitude	Number of individuals	Average coverage (%)
Lisboa	2013	Iberia	West	38.764	−9.250	10	91.2
Bizkaia	2013	Iberia	North	43.331	−2.782	10	99.0
Girona	2013	Iberia	Northeast	42.323	3.166	9	95.0
Granada	2013	Iberia	South	37.109	−4.170	8	85.0
Albacete	2013	Iberia	Centre–East	39.296	−2.069	9	97.2
Valladolid	2013	Iberia	Centre–North	41.581	−4.586	10	98.3
Valencia	2013	Iberia	East coast	39.409	−0.960	9	98.6
Vila Real	2009	Iberia	Northwest	41.300	−7.800	3	73.4
Devon	2011–2013	England	Southwest	50.552	−3.550	8	90.3
Dorset	2011	England	South–Centre	50.645	−2.315	7	71.7

### 2.2 Assessing exposure: environmental changes and ecological niche modelling

ENMs were generated using the maximum entropy modelling approach maxent v3.3.3 (Phillips, Anderson, & Schapire, 2006) to predict changes to the distribution of suitable conditions for *P. austriacus* under future climate change projections. Model extent was set as the whole of Europe to account for the full range of environmental conditions experienced by the species. Model resolution was set at ~1 km to match the resolution of the climatic data used in the sensitivity analysis. ENMs were built using 142 genetically confirmed nonclustered location records (Razgour et al., 2013) and included six climatic variables with future projections for 2070, a static topographic variable, slope and a land cover variable with no fine‐scale future projections (Table S1). We included land cover because such nonclimatic variables can greatly improve ENM predictive performance, even in the absence of future projections (Stanton, Pearson, Horning, Ersts, & Reşit Akçakaya, 2012). Future projections were carried out using the HadCM3_ES General Circulation Model (http://www.worldclim.org) and the IPCC5 + 8.5 W/m^2^ Representative Concentration Pathways scenario (IPCC 2013), representing the “worst‐case” scenario, whereby human consumption of fossil fuels is expected to remain the same as at present (Appendix S1 for further details on variable selection and model parameterization).

The predictive power of the ENMs was evaluated from ten cross‐validations, using the area under the receiver operator curve (AUC) criteria. Climatic suitability was determined based on predicted relative probability of occurrence and was averaged across the four cells adjacent to the population location to cover the colony home range (Razgour, Hanmer, & Jones, 2011). To calculate changes in range suitability within Iberia, continuous occurrence probability model outputs were reclassified into binary maps using the thresholding method that maximizes the sum of sensitivity and specificity (as recommended by Liu, White, & Newell, 2013).

Maximum temperatures and summer rainfall (Bio5 and Bio18, downloaded from WorldClim, http://www.worldclim.org) were used to test for environmental dissimilarity between current and future (2070) conditions. These variables represent climatic conditions that are predicted to change under future projections for Iberia (Diffenbaugh & Field, 2013) and are likely to affect bats. Increased aridity and prolonged droughts around the Mediterranean are predicted to affect insect prey availability during the summer (Frampton, Van Den Brink, & Gould, 2000), and thus decrease reproductive success in bats (Adams, 2010; Amorim, Matta, Beja, & Rebelo, 2015). In addition, bat survival in warmer and more arid conditions requires physiological adaptations to reduce evaporative water loss (Muñoz‐Garcia et al., 2016).

### 2.3 Assessing sensitivity: genomic data analysis

We generated a genomic data set containing thousands of anonymous genetic loci from across the *P. austriacus* genome using the reduced‐representation genome sequencing method double digest restriction‐site‐associated DNA sequencing, ddRADseq (Miller, Dunham, Amores, Cresko, & Johnson, 2007; Peterson, Weber, Kay, Fisher, & Hoekstra, 2012; library construction and sequencing protocols outlined in Appendix S1). Bioinformatics of the high‐throughput sequencing data was carried out using the STACKS pipeline (Catchen, Hohenlohe, Bassham, Amores, & Cresko, 2013; details in Appendix S1). To improve robustness of the data set, only RAD loci that contained fewer than three SNPs and were genotyped in at least 70% of the samples (67 individuals) were considered for analysis. The SNP data set was processed in plink v1.9 (Purcell et al., 2007) to remove individuals that had more than 50% missing data and loci with more than 30% missing data and minor allele frequencies below 0.03 (alleles present in less than three individuals). We also removed close relatives (based on identity‐by‐state distances, PI HAT >0.5) and loci that were out of Hardy–Weinberg equilibrium (*p* < .01) in more than two populations. Population‐level analyses were carried out on populations containing a minimum of seven individuals to ensure an adequate representation of allele frequencies (Willing, Dreyer, & van Oosterhout, 2012).

Genetic population structure was determined using individual‐based Bayesian assignment tests, implemented in the program structure v2.3.4 (Pritchard, Stephens, & Donnelly, 2000) (Appendix S1 for structure running procedures). The significance of genetic differences between populations and geographic regions (England vs. Iberia) was determined based on a multilocus analysis of molecular variance (amova) implemented in the r package gstudio (Dyer, 2009).

#### 2.3.1 Identifying a signature of climate‐driven adaptations

To identify a signature of climate‐driven local adaptations, we combined population genomics and ecological approaches. Outlier tests, as implemented in the programs Bayescan (Foll & Gaggiotti, 2008) and LOSITAN (FDist, Antao, Lopes, Lopes, Beja‐Pereira, & Luikart, 2008), were used to identify SNPs potentially under directional selection, or linked with genes under selection, based on higher levels of genetic differentiation among populations relative to expected neutral distributions (Appendix S1 for test parameters). Allele frequencies of SNPs identified as outliers were correlated against environmental variables (maximum temperature and summer rainfall) using logistic regressions (glm function in r), as described by Schoville, Widmer, Deschamps‐Cottin, and Manel (2013).

We carried out a genotype–environment association analysis to test for associations between allele frequencies and local environmental variables (maximum temperature and summer rainfall). We used the latent factor mixed model (lfmm) Frichot, Schoville, Bouchard, and Francois (2013) approach, implemented in the r package LEA (Frichot & François, 2015). We corrected for population structure through including the number of populations (*K*) identified by structure assignment tests as latent factors in the models. We performed five lfmm repetition runs with 1,000,000 iterations and 500,000 iterations for burn‐in. Z‐scores of multiple runs were combined using the median value, and *p*‐values were adjusted for expected FDR of 0.05 (following the procedures in Frichot & François, 2015; Appendix S2 for lfmm r script). SNPs that were found to be both under directional selection based on outlier tests and statistically associated with climatic variables based on the genotype–environment association analysis were classified as potentially associated with climate‐adaptive genetic variation, that is with adaptations to local climatic conditions. However, it is important to note that these SNPs may represent genomic regions linked to genes under selection rather than specific climate‐adaptive genes.

Genotype–environment associations between SNPs and climatic variables were investigated at two scales, across the whole study area (England and Iberia) and within Iberia, to account for clines in allele frequencies at neutral loci due to genetic drift and allele surfing during population expansion (Excoffier & Ray, 2008). The Iberian Peninsula acted as the main glacial refugium for *P. austriacus,* where a stable population was maintained across glacial cycles (Razgour et al., 2013). Hence, SNPs identified as potentially under selection within this area likely reflect true climate‐driven adaptations rather than artefacts of neutral processes that occurred during post‐glacial range expansion.

#### 2.3.2 Patterns of neutral genetic variation

Neutral genetic diversity was estimated based on levels of heterozygosity in the population after excluding SNPs identified as outliers (under selection) by Bayescan. We used the –het function in Plink to compare observed and expected individual levels of homozygosity. Heterozygosity was calculated as 1 − (mean population *F*), *F* being the coefficient estimation of observed (Obs) versus expected (Exp) homozygosity (Hom):

F=(Obs_Hom−Exp_Hom)/(Total−Exp_Hom).

### 2.4 Assessing range shift potential: landscape genetics analysis

Genetic distances between pairs of populations were estimated separately for the neutral SNP data set and for SNPs identified as a potentially under climate‐driven selection, using the *F*_st_ measure of genetic differentiation in the r package diversity (Keenan, McGinnity, Cross, Crozier, & Prodöhl, 2013). Geographic (Euclidean) distances between populations were calculated in arcgis v10 (ESRI). The analysis included landscape variables and resistance costs that were previously shown to affect functional connectivity in *P. austriacus* (Razgour, 2015; Razgour et al., 2014): habitat suitability measured through ENMs, forest cover variables, altitude and slope. We did not include landscape variables that were highly correlated with other variables or geographic distance (*R* ^2^ > .70) because they can lead to the identification of spurious inferences (Cushman, Wasserman, Landguth, & Shirk, 2013).

Landscape variables were converted to resistance cost surfaces in arcgis and were assigned resistance costs ranging from one (no resistance to movement) to 100 (strong barrier to movement). The sea was assigned a resistance cost of 200 to reflect the lower likelihood of bats crossing large expanses of water than land because previous studies have found limited gene flow across seas in this species (Razgour et al., 2014). We tested the effect of decreasing the resistance costs of crossing the sea to 120. We tested how changing the resistance costs of the different landscape variables and converting continuous into categorical variables affected the strength of the model associations with genetic differentiation (Appendix S1 for generating resistance cost surfaces).

circuitscape v4.0.5 (McRae, 2006) was used to calculate resistance distance matrices between populations and estimate potential movement pathways across the landscape based on the cumulative cost of movement due to landscape resistance. We used the nine populations as our focal nodes and selected the “pairwise” modelling mode (iterating across all population pairs in focal node file). Movement pathways (cumulative current maps) were generated based on present and future (2070) conditions to assess the future movement potential of individuals and adaptive genetic variation among populations.

We used multiple regressions on distance matrices (MRDM in the r package ecodist; Goslee & Urban, 2007) with 10,000 permutations to test for the effect of landscape variables on genetic differentiation (as a surrogate for gene flow and individual movement) between population pairs. We ran MRDM between *F*_st_ and all landscape variables and their different resistance costs to select the resistance cost combinations that showed the strongest correlations. Following Dyer, Nason, and Garrick (2010), we accounted for the effect of geographic distance using a stepwise approach. We first ran MRDM between *F*_st_ and geographic distance and then used the residuals from the regression as the response variable in subsequent MRDM models to test for associations with landscape variables. The best‐fit model was selected based on highest *R* ^2^ values and significant *p* values for all variables (*p* < .05). MRDM was also used to test whether genetic differentiation (*F*_st_) between populations in climate‐adaptive SNPs was a function of environmental dissimilarity (differences in maximum temperature and summer rainfall) between locations (isolation by environment).

### 2.5 Identifying level of risk

We developed a quantitative approach to identify the level of risk to populations from future climate change based on our three framework components, exposure, sensitivity and range shift potential. Assigned levels of risk aim to guide conservation prioritization and inform management decisions through highlighting which aspects should be the focus of conservation action.

Exposure was ranked from low (1) to high (4) based on changes in climatic suitability as predicted by the ENMs (reduction in relative probability of occurrence and changes from suitable to unsuitable conditions) and the extent of environmental dissimilarity between present and future conditions (Table 2).

**Table 2:** Variables and categories used to assess level of exposure to future changing climatic conditions. Formula indicates whether all variables were combined together or only one or two needed to be true. ENM refers to the outputs of the ecological niche model—continuous output for changes in relative occurrence probability, or binary output for changes in climatic suitability. Temperature and rainfall dissimilarity refer to differences between present and future (2070) conditions

Level of Exposure	Formula	ENM	Temperature dissimilarity	Rainfall dissimilarity
1 (low)	ENM + Temp + Rain	Change in relative occurrence probability <25%	Low: <6°C increase	Low: <25% decrease
		Area remains climatically suitable		
2 (medium–low)	ENM + (Temp OR Rain)	Change in relative occurrence probability >25%	Medium: 6–8°C increase	Medium: 25–50% decrease
		Area remains climatically suitable		
3 (medium–high)	ENM OR Temp OR Rain	Area changed from climatically suitable to unsuitable	High: >8°C increase	High: >50% decrease
4 (high)	ENM + (Temp OR Rain)	Area changed from climatically suitable to unsuitable	High: >8°C increase	High: >50% decrease

Sensitivity was determined based on the frequency of alleles in SNPs identified as potentially associated with warmer and drier climatic conditions (adaptive sensitivity), as well as overall levels of neutral genetic diversity (neutral sensitivity). Levels of adaptive sensitivity were determined based on the frequency of potential climate‐adaptive alleles in the population, looking at both overall mean frequencies across all loci (high [++ or +] <0.50; medium [0]–low [−] >0.50), and number of adaptive alleles present at particularly low frequencies (<0.25) in the population (Table 3). Levels of neutral sensitivity were assessed based on the potential contribution of neutral genetic diversity to future adaptive potential (− low sensitivity due to high levels of neutral genetic diversity; 0 medium sensitivity; + high sensitivity due to relatively low levels of genetic diversity). The two measures were combined together to give a single measure of overall sensitivity.

**Table 3:** Assessment of sensitivity based on the frequency of alleles identified as potentially associated with climate‐adaptive genetic variation in the population

Level of Sensitivity	Mean frequency across all adaptive loci	No. adaptive alleles at frequency <0.25
Very high (++)	<0.5	More than a third
High (+)	<0.5	Less than a third
Medium (0)	≥0.5	At least one
Low (−)	>0.5	None

Range shift potential was determined according to the degree of connectivity to other populations under future conditions or to areas predicted to be climatically suitable, as estimated based on Circuitscape movement density (cumulative current) maps. Populations were deemed to have low range shift potential if they were predicted to become isolated (low connectivity) under future conditions, or only connected to adjacent populations and climatically unsuitable areas.

The three framework components were combined together to determine the level of risk to Iberian populations of *P. austriacus*, ranging from low to high risk. When combining the three components, we placed higher weights on exposure to future changes in climatic conditions (Table 4).

**Table 4:** Integrating measures of exposure (Table 2), sensitivity (Table 3) and range shift potential to assess overall level of risk. Formula indicates whether all measures were combined together or only two needed to be true (Exp—Exposure, Sen—Sensitivity, Range—Range shift potential)

Risk Level	Formula	Exposure	Sensitivity	Range shift
Low	Exp + (Sen OR Range)	1	Low (−)	High (+)
Low	Exp + Sen + Range	2	Low (−)	High (+)
Medium	Exp + (Sen OR Range)	2	Mid (0)–high (+)	Low (−)
Medium	Exp + Sen + Range	3	Low (−)–mid (0)	High (+)
Medium–high	Exp + (Sen OR Range)	3–4	High (+/++)	Low (−)
High	Exp + Sen + Range	3–4	High (+/++)	Low (−)

## 3 RESULTS

### 3.1 Exposure to climate change

**Figure 3:**
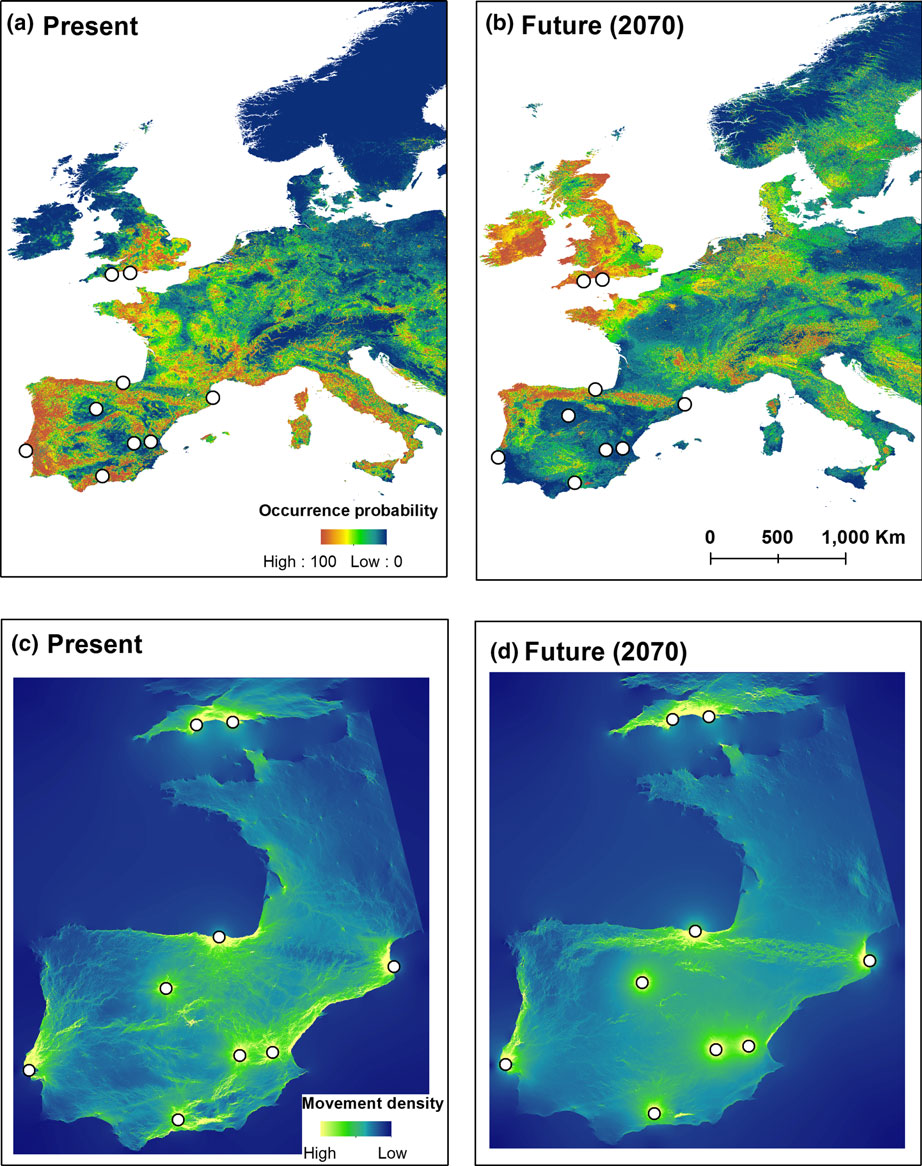
Predicted distribution of suitable conditions for *Plecotus austriacus* based on environmental niche models for present (a) and future (2070, b) conditions, and predicted movement density maps between populations based on landscape resistance due to present (c) and future (d) habitat suitability

ENMs had high discrimination and predictive abilities (AUC_train_ = 0.892, ACU_test_ = 0.821). The main variables affecting habitat suitability for *Plecotus austriacus* were winter and summer temperatures, summer rainfall and land cover (Fig. S1). Future models predicted 66.3% reduction in the suitable range of *P. austriacus* within Iberia. Under present conditions, 54.8% of Iberia was predicted to be climatically suitable, but only 18.5% was predicted to remain suitable by 2070. All populations were found within climatically suitable areas under present conditions. Under future conditions, five Iberian populations were predicted to occur either in climatically unsuitable areas (Albacete, Valencia and Girona) or in small isolated fragments of suitable habitats (Granada and Valladolid). Habitat suitability was predicted to increase slightly in England and around the north Iberian coast (Figure 3).

Analysis of environmental dissimilarity between current and future conditions predicted that the central and eastern populations (Valladolid, Albacete and Valencia) will experience the greatest increase in maximum temperatures (>7°C) and the greatest proportional reduction in summer rainfall (48–55%; Table 5). Based on ENM‐predicted changes in climatic suitability and the extent of environmental dissimilarity between present and future conditions, two populations were assigned high exposure values (Albacete and Valencia), two medium–high (Girona and Valladolid), one medium–low (Granada) and two low (Lisboa and Bizkaia; Table 6).

**Table 5:** Changes in climatic conditions (Tmax=maximum temperatures, Rain=summer rainfall) and climatic suitability (based on the ecological niche model (ENM)‐predicted occurrence probability) for *Plecotus austriacus* populations in Iberia and England. Locations where the greatest changes are predicted to occur (>8°C increase in maximum temperatures, >50% decrease in summer rainfall and change from suitable to unsuitable areas) are highlighted in grey

Population	*T*max (°C)	Rain (mm)	ENM (climatic suitability)	2070 *T*max (°C)	2070 Rain (mm)	2070 ENM	Change *T*max (°C)	Change Rain (mm)	% Change Rain	% Change ENM
Lisboa	25.3	36	100	28.6	28	80	+3.3	−8	−22.2	−20.0
Bizkaia	23.6	225	38	29.9	120	40	+6.3	−105	−46.7	+5.3
Girona	25.6	159	76	32.1	90	7	+6.5	−69	−43.4	−90.8
Granada	30.0	45	80	36.9	39	37	+6.9	−6	−13.3	−52.5
Albacete	31.6	61	34	40.4	27	17	+8.8	−34	−55.7	−50.0
Valladolid	29.3	65	56	38.4	34	41	+9.1	−31	−47.7	−26.8
Valencia	28.5	87	44	35.6	42	5	+7.1	−45	−51.7	−88.6
Devon	19.8	171	90	25.5	107	95	+5.7	−64	−37.4	+5.6
Dorset	20.8	164	60	27.4	98	63	+6.6	−66	−40.2	+5.0

**Table 6:** Identified level of risk to Iberian populations of *Plecotus austriacus* based on their extent of exposure to climate change (1 = low; 2 = medium; 3 = medium–high; 4 = high), overall sensitivity (+ high; 0 medium; − low), with sensitivity based on climatic adaptations and neutral genetic diversity in brackets, and range shift potential (+ high future connectivity; − low connectivity)

Population	Exposure	Sensitivity (adaptive; neutral)	Range shift	Risk level
Lisboa	1	− (−; 0)	+	Low
Bizkaia	1	+ (++;−)	+	Low
Granada	2	− (−; 0)	−	Medium
Girona	3	0 (+;−)	+	Medium
Valladolid	3	− (−;−)	−	Medium–high
Albacete	4	− (−;−)	−	Medium–high
Valencia	4	+ (++;−)	−	High

### 3.2 Assessing sensitivity to climate change

From the high‐throughput sequencing, we identified 39,825,843 demultiplexed, paired‐end reads, from which Stacks resolved 11,116 RAD tags that were present in at least 70% (*n* = 67) of all individuals screened and contained a maximum of three SNPs (average of 7719 ± 2474 tags per individual). After excluding individuals and SNPs with low coverage and removing SNPs with low minor allele frequencies, the final genotype data set contained 6067 SNPs scored from 83 individuals, belonging to 10 populations, with a total genotyping rate of 0.919. All populations had an average coverage >70% (Table 1). The population with the oldest samples, Vila Real, only contained three individuals with high enough coverage and was therefore removed from population‐level analyses, but the three individuals were retained in individual‐level analyses (structure and lfmm).

#### 3.2.1 Adaptive genetic variation

Bayescan identified 24 outlier SNPs potentially under selection. LOSITAN identified 224 SNPs as potentially under directional selection, which included 20 of the outlier SNPs also identified by Bayescan. Allele frequencies in 13 outlier SNPs were significantly correlated with either maximum temperatures (11 SNPs) or summer rainfall (10 SNPs). Significant correlations were also identified within Iberia between five SNPs and maximum temperatures and seven SNPs and summer rainfall (Table S2).

structure assignment tests divided the full data set into two main genetic clusters, separating the English and Iberian samples. The Iberian cluster was further divided into two clusters, separating the two northern populations, Bizkaia and Girona (Fig. S2). Therefore, lfmm was run with three latent factors for the full data set and two for Iberia. lfmm detected 93 outlier SNPs associated with maximum temperatures and 129 SNPs with summer rainfall across the study area. In the Iberia‐only data set, 177 SNPs were associated with maximum temperatures and 278 with summer rainfall. We identified eight SNPs potentially associated with climate‐adaptive genetic variation that were supported by all methods (Bayescan, lfmm and logistic regressions; Table S3). The spatial distribution of genetic variation in SNPs identified as potentially climate‐adaptive indicates a lower frequency of warm and dry adaptive alleles in the north and eastern Iberian populations (Bizkaia, Girona and Valencia; Fig. S3; Table S4), and therefore, these populations were classified as having high adaptive sensitivity to climate change.

Genetic differentiation in SNPs identified as potentially associated with climate‐adaptive genetic variation was high overall (mean *F*_st_ = 0.245 ± 0.15), but was substantially lower among the north and eastern Iberian populations and among the southern and western populations. Highest levels of differentiation were found between one English population (Devon) and most other populations, apart from the north Iberian populations (Table S5). Genetic differentiation in these climate‐adaptive SNPs was related to environmental dissimilarity between locations. Across the study area, genetic differentiation was correlated with dissimilarity in both maximum temperatures (MRDM: *R* ^2^ = .173, *F* = 7.1, *p* = .01) and summer rainfall (*R* ^2^ = .137, *F* = 5.4, *p* = .023), while within Iberia it was correlated with summer rainfall (*R* ^2^ = .228, *F* = 5.6, *p* = .028; Fig. S4).

#### 3.2.2 Neutral genetic variation

Genetic differentiation between populations based on the neutral data set ranged between 0.024 and 0.106 (mean *F*_st_ = 0.056 ± 0.03), with highest values between the English populations and all Iberian populations (Table S4). Genetic differences between populations (multilocus amova:* R* ^2^ = .490, *p* < .001) and regions (*R* ^2^ = .245, *p* < .001) were highly significant. Differences between populations remained significant within Iberia (*R* ^2^ = .279, *p* < .001).

Levels of neutral genetic diversity were lowest in the English populations, especially Dorset (heterozygosity = 0.664). Iberian populations had generally high levels of heterozygosity, with relatively lower levels in Granada (0.844) and Lisboa (0.885), and highest levels in Valencia, Bizkaia and Valladolid (all >0.95). Relative neutral genetic diversity was ranked from low (heterozygosity<0.75) to medium (0.75–0.9) and high (>0.9) (Table S6).

### 3.3 Determining range shift potential

Genetic differentiation in neutral markers across the study area was positively related to geographic distance (MRDM: *R* ^2^ = .649, *F* = 63.1, *p* = .0001) and to landscape resistance due to decreasing habitat suitability, as measured by the ENM (*R* ^2^ = .842, *F* = 180.7, *p* = .0001), decreasing forest cover (*R* ^2^ = .588, *F* = 48.6, *p* = .0001), increasing altitude (*R* ^2^ = .299, *F* = 14.5, *p* = .0004) and increasing slope (*R* ^2^ = .667, *F* = 68.2, *p* = .0001). The ENM showed the strongest correlations with genetic differentiation and was the only landscape variable that remained significant after accounting for geographic distance (*R* ^2^ = .197, *F* = 8.3, *p* = .005; Table S7). We obtained identical results when decreasing the resistance costs of dispersal over sea to 120 (Table S8).

Under present conditions, all Iberian populations showed high levels of landscape connectivity. Particularly high density of movement was predicted along the east coast of Iberia, connecting the southern and northeastern populations, and across the east‐to‐west central axis of the peninsula. Overall density of movement was predicted to decrease under future conditions, resulting in reduced connectivity between most populations and geographic areas. In particular, eastern (Valencia), central (Albacete and Valladolid) and southern (Granada) populations were predicted to become isolated and were therefore assigned low range shift potential. However, landscape connectivity was predicted to increase across the Pyrenees between the two northern populations and along the north Atlantic coast. Movement out of Iberia appears to be limited both under present and future conditions, but Iberia is predicted to become isolated under future conditions due to decreased habitat suitability in southern France (Figure 3).

### 3.4 Identifying populations under threat

When combining the effect of the three framework components, we identified one Iberian population (Valencia, east coast) at high risk due to high changes in climatic suitability (from suitable to unsuitable and high increases in maximum temperatures and reductions in summer rainfall), low frequency of SNPs identified as associated with climate‐adaptive genetic variation and limited future landscape connectivity. We identified two additional populations in the central regions (Albacete and Valladolid) that are of medium–high risk because despite high exposure to future changes and limited future connectivity, they have a relatively high frequency of adaptive genetic variation and high levels of neutral genetic diversity. In contrast, populations along the Atlantic coast (north and northwest of the peninsula) are likely to be of lower risk due to more limited changes in climatic suitability and either high future landscape connectivity or lower sensitivity (Table 6).

## 4 DISCUSSION

We propose an applied integrated framework to identify wildlife populations under threat from future climate change based on their extent of exposure to changing climatic conditions, inherent sensitivity due to identified signatures of adaptive and neutral genetic variation and range shift potential (Figure 1). Our framework aims to address an important challenge hampering conservation planning for species under climate change, the lack of inclusion of measures of sensitivity and adaptive capacity in assessments of vulnerability, which currently mainly focus on climate exposure (Butt et al., 2016). While previous studies discussed the importance of including sensitivity and adaptive capacity when assessing climate change vulnerability (e.g., Dawson et al., 2011; Pearson et al., 2014; Williams, Shoo, Isaac, Hoffmann, & Langham, 2008), this is the first study to directly incorporate empirical genomic data to quantify sensitivity and assess adaptive potential through the spread of adaptive genetic variation among populations. Moreover, unlike previous studies, we consider intraspecific vulnerability and population‐level responses to global climate change, an important but often neglected research priority in conservation biology.

### 4.1 Exposure to changing climatic conditions

To assess exposure to future climate change, we combined ENMs with a comparison of environmental dissimilarity between current and future conditions in key climatic variables that are likely to affect bat survival and reproductive success. This helped identify priority areas that are predicted to experience the greatest magnitude of change, the central regions and the Mediterranean coast. However, it is important to note that apart from north and northwest Atlantic coast areas, all Iberian populations are projected to experience maximum temperatures outside the current thermal range of the species. Indeed, the entire Iberian Peninsula is recognized as being under high threat from the effects of future climate change, and Mediterranean ecosystems are predicted to experience the greatest biodiversity changes in Europe due to the combined effect of climate and land use changes (Sala et al., 2000). In line with previous studies (Razgour et al., 2013), the ENM analysis predicts range contractions for *P. austriacus* across the southern part of its range, accompanied by expansion into more northern latitudes. The inclusion of land cover variables and finer‐scale resolution in the ENMs resulted in less severe projections of future range losses in Iberia, but greater projected losses in France, which will isolate the Iberian Peninsula.

Our assessment of exposure disregards the role of phenotypic plasticity or genetic adaptations in enabling populations to persist in areas predicted to experience climatic conditions outside the species’ current environmental niche (Hoffmann & Sgrò, 2011). However, evidence of niche conservatism in climatic tolerance suggests that this species may be unable to survive in climatically unsuitable areas in the future (Razgour et al., 2013).

### 4.2 Sensitivity due to adaptive and neutral genetic variation

Understanding adaptive genetic responses to environmental change in wild populations is essential for biodiversity conservation under global change. Monitoring adaptive responses can help identify populations and species that are not able to evolve fast enough to persist in rapidly changing environments, and suitable donor populations that can help increase adaptive potential through evolutionary rescue (Hansen, Olivieri, Waller, & Nielsen, 2012). Yet even though it is recognized that genetic variability is essential for the ability of species to adapt to environmental changes, genetic components are often neglected in future climate change studies (Pauls, Nowak, Bálint, & Pfenninger, 2013) and the genetic basis of evolutionary responses to climate change is still poorly understood (Franks & Hoffmann, 2012).

Advances in sequencing technologies have enabled genomic research on nonmodel organisms and wild populations and opened the door to identifying genetic features underlying local adaptations, thus advancing our understanding of natural selection and evolution (Hoban et al., 2016). However, sequencing costs are still prohibitively expensive when sampling a large number of individuals, particularly when a reference genome is not available (Narum, Buerkle, Davey, Miller, & Hohenlohe, 2013). Alternative approaches, such as ddRADseq, offer an affordable way of obtaining a genomewide perspective by targeting only a fraction of the genome, rendering them particularly suitable for answering ecological and conservation questions (Andrews et al., 2016). Such reduced‐representation techniques only sequence a small fraction of the genome, and therefore only offer an indication of available adaptive genetic variation (Lowry et al., 2017). Nevertheless, because RADseq provides a random sample of the genome, it is a powerful and efficient approach to study selection in natural populations and test for evidence of adaptive differentiation and its geographic distribution (Catchen et al., 2017).

Bats have been the subjects of several recent genomic studies, shedding light on the evolution of flight (Zhang et al., 2013) and echolocation (Parker et al., 2013). However, this is the first study to identify a signature of climate‐driven selection in bats. By combining population genomics and ecological approaches, we identified eight SNPs representing genomic regions that are potentially associated with climate‐adaptive genetic variation. While genetic differentiation in neutral SNPs was related to the effect of the landscape matrix on movement between populations, differentiation in climate‐adaptive SNPs was correlated with environmental dissimilarity between locations, indicating a pattern of isolation by environment as a result of local adaptations (Wang & Bradburd, 2014).

Adaptation to local environmental conditions is thought to involve subtle changes in allele frequencies because gene flow between populations can counteract local adaptations and the fixation of adaptive alleles (Rellstab, Gugerli, Eckert, Hancock, & Holderegger, 2015). These subtle changes, that is soft selective sweeps, are harder to detect by genome scans for outlier loci, especially when selection has not had sufficient time to substantially shift allele frequencies (Stapley et al., 2010). Approaches that are driven by ecological hypotheses (genotype–environment association analysis) are better able to detect ecologically relevant loci with small effects involved in local environmental adaptations (Joost et al., 2013). Because only population genomic approaches can detect complete selective sweeps, while ecological approaches are better suited for detecting subtle changes, combining both approaches is essential for obtaining a complete perspective on climate‐driven genetic adaptations. Ideally, where possible, these approaches should be combined with experimental testing and functional validation of fitness or a trait in the absence of the putative adaptive alleles, although such validation is still impossible for most experimental systems (Hoban et al., 2016).

Our framework focuses on an assessment of sensitivity to changes in climate based on genomic data. Sensitivity can also be assessed using experimental evolutionary approaches. Experimental studies measuring heritability of climate‐related traits in various plant species and *Drosophila* exposed to simulated climatic changes found that rates of evolution may be too slow to match predicted rates of future climate change (reviewed in Jump & Peñuelas, 2005). More recently, studies, primarily of plants, have combined genomic and experimental approaches to identify local adaptations and genes under climate‐driven selection based on differential fitness of geographically diverse ecotypes raised under common garden experiments (e.g., Fournier‐Level et al., 2011; Savolainen, Lascoux, & Merilä, 2013). However, such experimental approaches are not feasible for long‐lived organisms with long generation time and for many species of conservation concern, and results from such experiments may not always be relevant for natural populations (Bailey & Bataillon, 2016). Even in cases where an experimental approach was applied to animals, field common garden studies have largely failed to successfully incorporate fitness and genomic data (Savolainen et al., 2013). Therefore, in our framework we focus on the genomic approach, but acknowledge that sensitivity to climate change can be assessed using other approaches.

### 4.3 Landscape connectivity and range shift potential

Our framework applies primarily to relatively long‐lived vertebrates with long generation times and small population sizes, in which the rate of emergence and spread of novel adaptive alleles in populations through de novo mutations is likely to be too slow to respond to rapid future climate changes (Hoffmann & Sgrò, 2011). Therefore, we emphasize the role of landscape connectivity as an important component influencing the ability of populations to respond to future changes through the spread of adaptive alleles between populations.

Through combining landscape genetics with ENMs, we determined the effect of landscape connectivity on movement patterns and the ability of *P. austriacus* to respond to climate change by tracking changes to its environmental niche. We found that habitat suitability is the main barrier to movement across the western part of this species’ range. Using a predictive landscape genetics approach, we showed how changing niche suitability is likely to limit the future movement of individuals both within and out of Iberia. Species movement patterns are not only a function of external factors like landscape connectivity, but also of internal factors, like species’ movement capacity (Nathan et al., 2008). The maximum recorded dispersal distance in *P. austriacus*, 62 km (Riede, 2001), is insufficient for individuals from most Iberian populations to reach climatically suitable areas. Therefore, range shifts are more likely to be a gradual stepping stone process, involving the establishment of populations followed by further dispersal events. This highlights the importance of the availability of suitable habitats for range shifts in limited dispersal species.

Restricted future landscape connectivity will limit the movement of individuals between populations and consequently reduce the rate of evolutionary adaptation to changing conditions through reducing the spread of adaptive alleles among populations. Therefore, evolutionary rescue is unlikely without assisted translocation of individuals into populations with a low frequency of alleles associated with warm and dry conditions. However, even under high dispersal or translocation scenarios, evolutionary rescue in spatially structured populations may be impeded by local adaptations to heterogeneous environments that reduce the fitness of migrants carrying climate‐adaptive alleles (Schiffers, Bourne, Lavergne, Thuiller, & Travis, 2013). This further strengthens the urgent need for an integrated framework to identify populations at high risk and suitable donor populations based on patterns of adaptation to local environmental conditions.

## 5 CONCLUSIONS

We developed an integrated framework to assess vulnerability to future climate change. We demonstrate how our framework can be applied to vertebrates with relatively limited dispersal abilities through combining genomic data with ENMs, spatial analysis and a predictive landscape genetics approach under a risk assessment framework. Our study highlights the importance of incorporating ecological and genomic data to predict both the sensitivity of populations to future changes and their ability to shift their distribution to track changes in environmental suitability. As evolutionary rescue in most vertebrates and species of conservation concern is more likely to occur through the movement of individuals with adaptive alleles between populations (Vander Wal, Garant, Festa‐Bianchet, & Pelletier, 2013), understanding movement ecology and limits to future landscape connectivity is essential for predicting the ability of populations to persist under climate change.

Assigned levels of threat can help prioritize and inform conservation action under climate change. Conservation management can focus on either rescuing high‐risk populations (through translocation of the entire population or of individuals with relevant adaptive variation into the population) or increasing landscape connectivity to facilitate range shifts and the spread of adaptive genetic variation to reduce threats to medium‐ and medium–high‐risk populations. As such, our framework can contribute to transforming conservation management under climate change from a crisis‐driven response to more anticipatory and predictive measures (Gillson, Dawson, Jack, & McGeoch, 2013).

## AUTHOR CONTRIBUTION

O.R. conceived and designed the study. K.P. and G.J. advised on study design. O.R., J.J., C.I., H.R. and A.A. collected the genetic samples. O.R. and J.B.T. carried out the molecular laboratory work and performed the data analysis. S.M. advised on data analysis. O.R. wrote the first draft of the manuscript, and all authors contributed to revisions.

## Data Availability

The raw sequence data from this study have been submitted to the EBI European nucleotide archive under project number PRJEB21291 (see Table S9 for barcode sample identifiers). SNP data set in Genepop and Map/PED formats, structure input and output files and Maxent output files – Data available from the Dryad Digital Repository: https://doi.org/10.5061/dryad.kv4g1 (https://doi.org/10.5061/dryad.kv4g1). r scripts used in the analysis: online Supporting Information.
