# Supplementary material for: An integrated framework to identify wildlife populations under threat from climate change
Source: Mol Ecol Resour. 2017 Jul 25;18(1):18–31. doi: 10.1111/1755-0998.12694 (PMC6849758; doi:10.1111/1755-0998.12694)
Supplement: Supplementary file 1 [file MEN-18-18-s001.pdf]

MOLECULAR ECOLOGY RESOURCES

Supplemental Information for:

An integrated framework to identify wildlife populations under threat from climate change

Orly Razgour, John B. Taggart, Stephanie Manel, Javier Juste, Carlos Ibáñez, Hugo Rebelo, Antton Alberdi, Gareth Jones, Kirsty Park

Table of Contents:

**Appendix 1 - Supplemental Methods**	Pages 2-7
**Appendix 2 - LFMM R script**	Pages 8-9
**Appendix 3 - Supplemental Tables**	Pages 10-20
**Appendix 4 - Supplemental Figures**	Pages 21-24

MOLECULAR ECOLOGY RESOURCES

Appendix 1 – Supplementary Methods

Ecological niche modelling procedures

We addressed collinearity in predictor variables through removing strongly correlated variables (R > 0.75; calculated in ENMTools, Warren *et al.* 2010), as well as variables that did not contribute to the model. Over-fitting and the effect of adjusting model parameters and regularization were assessed using Akaike Information Criterion (AIC) as implemented in ENMTools. The best fit model based on AIC scores included a regularization value of one, five features (linear, quadratic, product, threshold and hinge) and 1500 iterations. Following the recommendations in Merow *et al.* (2013), we used the raw output during the model comparison stage and the cumulative output to produce our final models and for model evaluation.

Molecular lab protocols

Genomic DNA was extracted using the Qiagen DNeasy Blood and Tissue extraction kit, and quantified with Qubit® Fluorometer 2.0 and the dsDNA High Sensitivity assay kit (Invitrogen, ThermoFisher Scientific). DNA degradation was assessed through visualisation on1% agarose gels. We excluded samples with low DNA quantity (<2 ng/µl) or quality. To equalise DNA concentration at around 2-3 ng/µl, we diluted samples with high DNA concentrations using the Qiagen elution buffer (EB).

The ddRAD library preparation protocol was based on the methodology originally reported by Peterson *et al*. (2012), with modifications / refinements as described in Manousaki *et al*. (2016). Briefly, each of the 95 DNA samples was simultaneously digested by two high fidelity restriction enzymes (RE): SbfI (CCTGCA|GG recognition site), and SphI (GCATG|C recognition site), both sourced from New England Biolabs (NEB, UK). Digestions were incubated at 37°C for 50 min, using 10 U of each enzyme per microgram DNA in 1× CutSmart Buffer (NEB), in a 6 µL total reaction volume. After cooling the reactions to room temperature, 3 µL of a premade barcode / adapter mix was added to the digested DNA, and incubated at room temperature for 10 min. This adapter mix comprised individual-specific barcoded combinations of P1 (SbfI-compatible) and P2 (SphI-compatible) adapters at 6 nM and 72 nM concentrations respectively, in 1× reaction buffer 2 (NEB). Adapters were compatible with Illumina sequencing chemistry (see Peterson *et al*., 2012). The barcoded adapters were designed such that adapter– genomic DNA ligations did not reconstitute RE sites, while residual RE activity limited concatemerization of genomic fragments. The adapters included an inline five- or seven-base barcode for sample identification. Ligation was performed over 3 hr at 22°C by addition of a further 3 µL of a ligation mix comprising 4 mM rATP (Promega, UK), and 2000 cohesive-end units of T4 ligase (NEB) in 1× CutSmart buffer. The ligated samples were then heat denatured at 65°C for 20 min, cooled, and combined into a single pool. The pooled sample was column-purified (MinElute PCR Purification Kit, Qiagen, UK), and eluted in 100 µL EB buffer (Qiagen, UK). Size selection of fragments, ranging from approximately 400 bp to 700 bp, was performed by agarose gel separation. Following gel purification (MinElute Gel Extraction Kit, Qiagen, UK), the eluted size-

selected template DNA (60 µL in EB buffer) was PCR amplified (13 cycles PCR; 28 separate 12.5-µL reactions, each with 1 µL template DNA) using a high fidelity Taq polymerase (Q5 Hot Start High-Fidelity DNA Polymerase, NEB). The PCR reactions were combined (350 µL total), and column-purified (MinElute PCR Purification Kit). The 55 µL eluate, in EB buffer, was then subjected to a further size-selection cleanup using an equal volume of AMPure magnetic beads (Perkin-Elmer, UK), to maximize removal of small fragments (less than ca. 200 bp). The final library was eluted in 19 µL EB buffer and sequenced, over three consecutive Illumina MiSeq runs (v2 chemistry, 300 cycle kit, 162 bp paired end reads; Illumina, Cambridge, UK).

Bioinformatics for genomic data analysis

The MiSeq generated reads were processed using a software pipeline designed specifically for RAD analysis, Stacks (v.1. 17; Catchen *et al*., 2013). First, the ‘process_radtags’ function was used to demultiplex the individual samples. During this step sequence reads with quality scores below 10, missing either restriction site or with ambiguous barcodes were discarded. Barcodes were removed and all sequences trimmed to be no greater than 148 bases long. For the purposes of this analysis paired-end reads were treated as separate loci, read 2 sequences being appended to read 1 sequence files. These sequences were assigned to RAD loci and genotypes using the ‘denovo_map.pl’ component of Stacks. The key parameter values employed in identifying RAD loci were; a minimum stack depth of 10 (m=10), a maximum of 2 mismatches allowed in a locus (M=2) in an individual and up to 1 mismatch between loci when building the catalog (n=1). Finally the ‘populations’ component of Stacks was used to export

filtered data (polymorphic loci containing 1-3 SNPs and present in at least 70% of samples for each population) in PLINK file format (PED and MAP files).

Population structure analysis methods

We ran assignment tests in STRUCTURE (Pritchard *et al.* 2000) for population cluster values of K=1-9. We performed five independent runs for each K with 100,000 burn-in iterations followed by 500,000 MCMC steps. We assumed the admixture model and did not include any prior information on populations. The number of distinct clusters was determined using STRUCTURE HARVESTER (Earl & von Holdt 2012) based on the conservative Evanno's method (Evanno *et al.* 2005). We re-ran the analysis for each identified population cluster to look for sub-structuring. Cluster assignment was visualised with DISTRUCT (Rosenberg 2004).

Outlier scan methods

Bayescan (Foll & Gaggiotti 2008) was run with 1,000,000 iteration, 50,000 burn-in and 20 pilot runs. Results were visualised in R using the script provided with the Bayescan download package with no modifications, setting false discovery rate (FDR) to 0.05.

LOSITAN (Antao *et al*. 2008) was run with 1,000,000 simulations under the Infinite Alleles mutation model, using the 'Neutral' mean Fst and forcing mean Fst options, and setting FDR to 0.05 and confidence intervals to 0.99.

Methods for generating resistance cost surfaces for the landscape genetics analysis

The ENM was transformed into opposite continuous resistance costs, whereby high probability of occurrence (100) received the lowest resistance costs (1), as well as into a categorical layer divided into 10 quantiles. The altitude and slope layers (downloaded from WordlClim, www.worldclim.org) were converted into continuous resistance costs that increased with elevation/slope (range 0-100). The forest cover gradient variable was generated through reclassifying the GlobCover2009 map into five forest cover categories. We also generated a continuous tree cover variable from Hansen *et al*. (2013) percent tree canopy cover map.

References

Antao T, Lopes A, Lopes RJ, Beja-Pereira A, Luikart G (2008) LOSITAN: a workbench to detect molecular adaptation based on a Fst-outlier method. *BMC Bioinformatics*, **9**, 323.

Catchen J, Hohenlohe P, Bassham S, Amores A, Cresko W (2013) Stacks: an analysis tool set for population genomics. *Molecular Ecology,* **22**, 3124–3140.

Earl DA, von Holdt BM (2012) STRUCTURE HARVESTER: a website and program for visualizing STRUCTURE output and implementing the Evanno method. *Conservation Genetics Resour*ces, **4**, 359–361.

Evanno G, Regnaut S, Goudet J (2005) Detecting the number of clusters of individuals using the software structure: a simulation study. *Molecular Ecology*, **14**, 2611–2620.

Foll M, Gaggiotti OE (2008) A genome scan method to identify selected loci appropriate for both dominant and codominant markers: A Bayesian perspective. *Genetics*, **180**, 977–993.

Hansen MC, Potapov PV, Moore R *et al*. (2013) High resolution global maps of 21st century forest cover change. *Science*, **342**, 850–853.

Manousaki T, Tsakogiannis A, Taggart JB, *et al*. (2016) Exploring a non-model teleost genome through RAD sequencing - Linkage mapping in Common Pandora, *Pagellus erythrinus* and comparative genomic analysis. *G3 Genes Genomes Genetics,* **6**, 509–519.

Merow C, Smith MJ, Silander JA (2013) A practical guide to MaxEnt for modeling species' distributions: what it does, and why inputs and settings matter. *Ecography*, **36**, 1058–1069.

Peterson BK, Weber JN, Kay EH, Fisher HS, Hoekstra HE (2012) Double digest RADseq: an inexpensive method for de novo SNP discovery and genotyping in model and non-model species. *PLoS ONE*, **7**(5), e37135.

Pritchard JK, Stephens M, Donnelly P (2000) Inference of population structure using multilocus genotype data. *Genetics*, 1**55**, 945–959.

Rosenberg NA (2004) DISTRUCT: a program for the graphical display of population structure. *Molecular Ecology Notes*, **4**, 137–138.

Warren DL, Glor RE, Turelli M (2010) ENMTools: a toolbox for comparative studies of environmental niche models. *Ecography*, **33**, 607–611.

MOLECULAR ECOLOGY RESOURCES

Appendix 2 – LFMM R script

```
## Script for running LFMM through the R package LEA (Frichot & François 2015) based on the
tutorial in http://membres-timc.imag.fr/Olivier.Francois/LEA/tutorial.htm

library(LEA)

# Data input from ped file
data = read.table("snp_dataset.ped")
output = ped2lfmm("snp_dataset.ped")

# Input environmental data from text file saved as '.env'
data_env = read.table("rainwarmq.env")

# Running lfmm
project = NULL
project = lfmm("snp_dataset.lfmm", "rainwarmq.env", K = 3, repetitions = 5, iterations =
100000, burnin = 10000, CPU = 4,
          project = "new")

summary(project)

# Get the zscores of each run for K = 3
zs_k3_rain = z.scores(project, K = 3)

# Combine the z-scores using the Stouffer method
zs.stouffer_k3_rain = apply(zs_k3_rain, MARGIN = 1, median)

# Calculate the genomic inflation factor
lambda_k3_rain = median(zs.stouffer_k3_rain^2)/.456

# Calculate adjusted p-values
cp.values_k3_rain = pchisq(zs.stouffer_k3_rain^2/lambda_k3_rain, df = 1, lower = FALSE)
plot(ecdf(cp.values_k3_rain),xlim=c(0,1),ylim=c(0,1))
abline(a=0,b=1)

for (alpha in c(.05,.1,.15,.2)) {
  # expected FDR
  print(paste("expected FDR:", alpha))
  L = length(cp.values_k3_rain)
```

```
# Return a list of candidates with an expected FDR of alpha.
  w = which(sort(cp.values_k3_rain) < alpha * (1:L) / L)
  candidates = order(cp.values_k3_rain)[w]

  # Estimated FDR and True Positives
  estimated.FDR_k3_rain = length(which(candidates <= 350))/length(candidates)
  estimated.TP_k3_rain = length(which(candidates > 350))/50
  print(paste("FDR:", estimated.FDR_k3_rain, "True Positive:", estimated.TP_k3_rain))
}

# Get list of candidate loci using the Benjamini-Hochberg procedure
q = 0.05       # expected false discovery rate
L = length(cp.values_k3_rain)
a <-sort(cp.values_k3_rain)
plot(a)
lines(q*(1:L)/L)
w = which(sort(cp.values_k3_rain) < q * (1:L) / L)
candidates_k3_rain_0.05 = order(cp.values_k3_rain)[w]
print(candidates_k3_rain_0.05)

# Get the p-values
p_k3_rain = p.values(project, K = 3)

# Get the -log10(p-values) for K = 3
mp_k3_rain = mlog10p.values(project, K = 3)

# Write as text files
write.table(zs.stouffer_k3_rain, "zs_k3_rain.txt", sep="\t")
write.table(mp_k3_rain, "log10_k3_rain.txt", sep="\t")
write.table(p_k3_rain, "p_values_k3_rain.txt", sep="\t")
write.table(candidates_k3_rain_0.05, "candidate_loci_k3_rain_0.05.txt", sep="\t")
```

MOLECULAR ECOLOGY RESOURCES

Appendix 3 – Supplemental Tables

Table S1 – Environment variables included in the ecological niche models and their source.

Variable	Source	Comments
Bio7 - Temperature annual range	WorldClim (www.worldclim.org)	
Bio10 - Mean temperature of the warmest quarter	WorldClim (www.worldclim.org)	
Bio11 - Mean temperature of the coldest quarter	WorldClim (www.worldclim.org)	
Bio12 - Annual rainfall	WorldClim (www.worldclim.org)	
Bio15 - Rainfall seasonality	WorldClim (www.worldclim.org)	
Bio18 - Rainfall during the warmest quarter	WorldClim (www.worldclim.org)	
Slope	WorldClim (www.worldclim.org)	
Land cover - GlobCover 2009 map	European Space Agency (http://due.esrin.esa.int/page_globcover.php)	reclassified into 10 categories

MOLECULAR ECOLOGY RESOURCES

Table S2 – *Plecotus austriacus* SNPs identified by Bayescan outlier test as potentially under selection and the significance of their correlations (logistic regressions) with summer rainfall (GLMs rain) and temperature (GLMs temp) across the whole study areas (Full) and within Iberia (IB). Significant correlations after Bonferroni correction are highlighted in bold.

Outlier SNPs	GLMs rain full	GLMs rain IB	GLMs temp full	GLMs temp IB
517	NS	NS	**P<0.001**	NS
1268	**P<0.001**	**P<0.001**	NS	NS
1644	**P<0.01**	P<0.05	NS	P<0.05
1911	NS	NS	**P<0.001**	NS
2196	zero inflated	zero inflated	zero inflated	zero inflated
3848	**P<0.01**	NS	**P<0.001**	P<0.05
3869	zero inflated	zero inflated	zero inflated	zero inflated
4735	NS	NS	**P<0.01**	NS
5401	zero inflated	zero inflated	zero inflated	zero inflated
5874	**P<0.001**	**P<0.001**	NS	NS
7135	NS	NS	NS	**P<0.001**
9060	zero inflated	zero inflated	zero inflated	zero inflated
9306	zero inflated	zero inflated	zero inflated	zero inflated
9872	NS	**P<0.01**	NS	NS
10617	**P<0.001**	**P<0.001**	P<0.01	NS
12222	**P<0.01**	NS	**P<0.001**	P<0.05
14219	zero inflated	zero inflated	zero inflated	zero inflated
14883	**P<0.01**	NS	**P<0.001**	P<0.05
15650	**P<0.001**	**P<0.01**	**P<0.001**	**P<0.01**
18702	P<0.01	NS	P<0.01	P<0.01
22104	P<0.01	NS	P<0.01	P<0.01
22863	NS	**P<0.01**	NS	NS
23049	zero inflated	zero inflated	zero inflated	zero inflated
24071	NS	NS	NS	NS

MOLECULAR ECOLOGY RESOURCES

Table S3 – The eight *Plecotus austriacus* SNPs identified as under climate-driven selection by Bayescan, LFMM, logistic regressions (GLMs) and LOSITAN (FDist), with their associated climatic variables and LFMM dataset (Full = whole study area; IB = Iberia only).

SNP name	Climate variable	LFMM dataset	GLMs	LOSITAN	Comments
**1268**	Rain	IB	P<0.001	P<0.01	
**1644**	Rain	Full + IB	P<0.001	P<0.005	
**3848**	Temperature	IB	P<0.001	P<0.005	
**5874**	Rain	IB	P<0.001	P<0.01	
**9872**	Rain	IB	P<0.01	P<0.005	
**10617**	Temperature + Rain	Full + IB	P<0.001	NS	Full only for Rain
**14883**	Temperature + Rain	IB	P<0.001	P<0.005	
**15650**	Temperature + Rain	Full + IB	P<0.001	P<0.005	Full only for Rain

MOLECULAR ECOLOGY RESOURCES

Tables S4 –Frequencies of potential climate-adaptive alleles in the Iberian *Plecotus austriacus* populations, including overall mean frequencies. Sensitivity is determined based on the overall mean frequency of potential climate-adaptive alleles in the population (high sensitivity <0.5, low sensitivity >0.5) and the number of adaptive alleles present at particularly low frequencies (<0.25), ranging from very high (++), to high (+), medium (0), and low (-).

SNP	Lisboa	Bizkaia	Girona	Granada	Albacete	Valladolid	Valencia
1268	1.00	0.50	0.33	1.00	0.89	0.60	0.50
1644	0.75	0.50	0.28	0.58	0.89	0.50	0.56
3848	0.72	0.65	0.50	0.79	1.00	0.72	0.78
5874	0.94	0.50	0.33	1.00	0.89	0.60	0.50
9872	0.85	0.15	0.61	0.31	0.44	0.30	0.17
10617	0.94	0.10	0.44	0.67	0.62	0.69	0.28
14883	0.31	0.30	0.20	0.69	0.56	0.50	0.06
15650	0.50	0.19	0.29	0.43	0.72	0.80	0.19
**Mean**	0.75	0.36	0.37	0.68	0.75	0.59	0.38
**Sensitivity**	-	++	+	-	-	-	++

MOLECULAR ECOLOGY RESOURCES

Table S5 – Genetic differentiation (Fst values) between *Plecotus austriacus* populations based on the neutral dataset (top triangle) and SNPs potentially associated with climate-adaptive genetic variation (bottom triangle). Lis=Lisboa, Bas= Bizkaia, Cat=Girona, Dev=Devon, Dor=Dorset, Gra=Granada, Alb=Albacete, Vld=Valladolid, and Val=Valencia.

	**Lis**	**Bas**	**Cat**	**Dev**	**Dor**	**Gra**	**Alb**	**Vld**	**Val**
**Lis**		0.038	0.035	0.106	0.091	0.027	0.035	0.027	0.036
**Bas**	0.350		0.026	0.088	0.078	0.031	0.038	0.032	0.041
**Cat**	0.287	0.026		0.080	0.070	0.029	0.034	0.025	0.033
**Dev**	0.508	0.298	0.266		0.057	0.100	0.106	0.097	0.101
**Dor**	0.292	0.288	0.097	0.541		0.088	0.094	0.082	0.093
**Gra**	0.042	0.285	0.272	0.476	0.333		0.028	0.024	0.033
**Alb**	0.074	0.288	0.296	0.559	0.399	0.039		0.028	0.037
**Vld**	0.080	0.203	0.110	0.406	0.195	0.078	0.061		0.031
**Val**	0.319	0.001	0.022	0.334	0.234	0.302	0.277	0.195	

MOLECULAR ECOLOGY RESOURCES

Table S6 – Measure of heterozygosity in neutral SNPs and corresponding relative levels of neutral genetic diversity in *Plecotus austriacus* populations. Relative neutral genetic diversity is ranked from low (Het<0.75) to medium (Het=0.75-0.9) and high (Het>0.9). Neutral sensitivity to future climate change is determined based on the potential contribution of genetic diversity to future adaptive potential (- low sensitivity due to high levels of neutral genetic diversity; 0 medium sensitivity; + high sensitivity due to relatively low levels of genetic diversity).

Population	Heterozygosity	Neutral Genetic Diversity	Neutral Sensitivity
**Lisboa**	0.8855	medium	0
**Bizkaia**	0.9638	high	-
**Girona**	0.9182	high	-
**Devon**	0.8351	medium	0
**Dorset**	0.6636	low	+
**Granada**	0.8442	medium	0
**Albacete**	0.9436	high	-
**Valladolid**	0.9596	high	-
**Valencia**	0.9711	high	-

MOLECULAR ECOLOGY RESOURCES

Tables S7 – Results of multiple regressions on distance matrices (MRDM) of genetic distance (Fst) in the *Plecotus austriacus* neutral dataset against geographic distance (geo-dist) and landscape variables for which the sea was assigned a cost of 200 (ENM=ecological niche model, forest=forest cover gradient), and of the residuals of the regression of genetic and geographic distance (Fst~geo-dist) against landscape resistance.

Variables	R^2	F	P	Comments
Fst ~ geo-dist	0.649	63.1	0.0001	
Fst ~ ENM continuous	0.842	180.7	0.0001	
Fst ~ ENM 10 quantiles	0.798	134.5	0.0001	
Fst ~ forest cover gradient	0.588	48.6	0.0001	
Fst ~ % tree cover continuous	0.572	45.4	0.0001	
Fst ~ altitude	0.299	14.5	0.0004	
Fst ~ slope	0.667	68.2	0.0001	
Fst ~ ENM + forest	0.844	89.1	0.0001	forest NS
Fst ~ ENM + altitude	0.85	93.6	0.0001	altitude NS
Fst ~ ENM + slope	0.842	88.3	0.0001	slope NS
Fst ~ ENM + forest + altitude	0.851	60.9	0.0001	forest/altitude NS
Residuals(Fst~geo-dist) ~ ENM	0.197	8.3	0.005	
Residuals(Fst~geo-dist) ~ forest	NS		0.181	NS
Residuals(Fst~geo-dist) ~ altitude	NS		0.831	NS
Residuals(Fst~geo-dist) ~ slope	NS		0.884	NS
Residuals(Fst~geo_dist) ~ ENM + forest	NS		0.614	NS
Residuals(Fst~geo_dist) ~ ENM + altitude	NS		0.304	NS
Residuals(Fst~geo-dist) ~ ENM + slope	NS		0.184	NS

Tables S8 – Results of multiple regressions on distance matrices (MRDM) of genetic distance (Fst) in the *Plecotus austriacus* neutral dataset against geographic distance (geo-dist) and landscape variables for which the sea was assigned a cost of 120 (ENM=ecological niche model, forest=forest cover gradient), and of the residuals of the regression of genetic and geographic distance (Fst~geo-dist) against landscape resistance.

Variables	R^2	F	P	Comments
Fst ~ geo_dist	0.649	63.1	0.0001	
Fst ~ ENM	0.715	85.4	0.0001	
Fst ~ forest	0.588	48.6	0.0001	
Fst ~ altitude	NS		0.0921	NS
Fst ~ slope	0.503	34.4	0.0001	
Fst ~ ENM + forest	0.757	51.4	0.0002	
Fst ~ ENM + altitude	0.754	50.5	0.0001	
Fst ~ ENM + slope	0.727	43.9	0.0001	slope NS
Fst ~ ENM + forest + altitude	0.776	36.9	0.0001	forest/altitude NS
Residuals(Fst~geo_dist) ~ ENM	0.172	7.1	0.01	
Residuals(Fst~geo_dist) ~ forest	NS		0.355	NS
Residuals(Fst~geo_dist) ~ slope	NS		0.632	NS
Residuals(Fst~geo_dist) ~ altitude	NS		0.197	NS
Residuals(Fst~geo_dist) ~ ENM + forest	NS		0.813	NS
Residuals(Fst~geo_dist) ~ ENM + slope	NS		0.567	NS
Residuals(Fst~geo_dist) ~ ENM + altitude	NS		0.544	NS

Table S9 – Combinatorial barcodes used to identify individual bat samples in the pooled ddRAD sequence output. Read 1 sequences begin with P1 barcode; paired Read 2 sequences begin with P2 barcode. Detailed location information is given in Table 1.

#	Sample ID	Population	P1 barcode (read 1)	P2 barcode (read 2)
1	Bas_001	Basque	TCTCTCA	TAGCA
2	Bas_003	Basque	GTACACA	TAGCA
3	Bas_005	Basque	CTCTTCA	TAGCA
4	Bas_006	Basque	CTAGGAC	TAGCA
5	Bas_008	Basque	ACGTA	TAGCA
6	Bas_009	Basque	AGAGT	TAGCA
7	Bas_010	Basque	ATGCT	TAGCA
8	Bas_011	Basque	GACTA	TAGCA
9	Bas_012	Basque	CAGTCAC	TAGCA
10	Bas_013	Basque	GCTAACA	TAGCA
11	Cat_017	Catalunya	ACACGAG	TAGCA
12	Cat_019	Catalunya	AGGACAC	TAGCA
13	Cat_021	Catalunya	TCAGA	AGCTGTC
14	Cat_023	Catalunya	GATCG	AGCTGTC
15	Cat_024	Catalunya	CATGA	AGCTGTC
16	Cat_026	Catalunya	ATCGA	AGCTGTC
17	Cat_027	Catalunya	TCGAG	AGCTGTC
18	Cat_028	Catalunya	GTCAC	AGCTGTC
19	Cat_029	Catalunya	GCATT	AGCTGTC
20	Cat_031	Catalunya	CGATA	AGCTGTC
21	Aqu_P02	Lisboa	TGCAACA	AGCTGTC
22	Aqu_P03	Lisboa	CGTATCA	AGCTGTC
23	Aqu_P04	Lisboa	CACAGAC	AGCTGTC
24	Aqu_P05	Lisboa	ACTGCAC	AGCTGTC
25	Aqu_P06	Lisboa	TCTCTCA	AGTCA
26	Aqu_P07	Lisboa	GTACACA	AGTCA
27	Aqu_P08	Lisboa	CTCTTCA	AGTCA
28	Aqu_P09	Lisboa	CTAGGAC	AGTCA
29	Aqu_P11	Lisboa	ACGTA	AGTCA
30	Aqu_P12	Lisboa	AGAGT	AGTCA

MOLECULAR ECOLOGY RESOURCES

Table S9 continued

#	Sample ID	Population	P1 barcode (read 1)	P2 barcode (read 2)
31	Pal_051	Albacete	ATGCT	AGTCA
32	Pal_052	Albacete	GACTA	AGTCA
33	Pal_053	Albacete	CAGTCAC	AGTCA
34	Pal_054	Albacete	GCTAACA	AGTCA
35	Pal_055	Albacete	ACACGAG	AGTCA
36	Pal_056	Albacete	AGGACAC	AGTCA
37	Pal_057	Albacete	TCAGA	TACGTGT
38	Pal_058	Albacete	GATCG	TACGTGT
39	Pal_059	Albacete	CATGA	TACGTGT
40	Pal_060	Albacete	ATCGA	TACGTGT
41	Gra_071	Granada	TCGAG	TACGTGT
42	Gra_072	Granada	GTCAC	TACGTGT
43	Gra_073	Granada	GCATT	TACGTGT
44	Gra_074	Granada	CGATA	TACGTGT
45	Gra_075	Granada	TGCAACA	TACGTGT
46	Gra_076	Granada	CGTATCA	TACGTGT
47	Gra_077	Granada	CACAGAC	TACGTGT
48	Gra_078	Granada	ACTGCAC	TACGTGT
49	Gra_079	Granada	TCTCTCA	GCATA
50	Val_081	Valladoid	GTACACA	GCATA
51	Val_082	Valladoid	CTCTTCA	GCATA
52	Val_083	Valladoid	CTAGGAC	GCATA
53	Val_085	Valladoid	ACGTA	GCATA
54	Val_086	Valladoid	AGAGT	GCATA
55	Val_087	Valladoid	ATGCT	GCATA
56	Val_088	Valladoid	GACTA	GCATA
57	Val_090	Valladoid	CAGTCAC	GCATA
58	Val_091	Valladoid	GCTAACA	GCATA
59	Val_092	Valladoid	ACACGAG	GCATA

MOLECULAR ECOLOGY RESOURCES

Table S9 continued

#	Sample ID	Population	P1 barcode (read 1)	P2 barcode (read 2)
60	Val_094	Valencia	AGGACAC	GCATA
61	Val_095	Valencia	TCAGA	GAGATGT
62	Val_096	Valencia	GATCG	GAGATGT
63	Val_097	Valencia	CATGA	GAGATGT
64	Val_098	Valencia	ATCGA	GAGATGT
65	Val_099	Valencia	TCGAG	GAGATGT
66	Val_100	Valencia	GTCAC	GAGATGT
67	Val_101	Valencia	GCATT	GAGATGT
68	Val_103	Valencia	CGATA	GAGATGT
69	Des_104	Vila Real	TGCAACA	GAGATGT
70	Des_105	Vila Real	CGTATCA	GAGATGT
71	Des_106	Vila Real	CACAGAC	GAGATGT
72	Des_107	Vila Real	ACTGCAC	GAGATGT
73	Des_108	Vila Real	TCTCTCA	CGATC
74	Des_109	Vila Real	GTACACA	CGATC
75	Des_110	Vila Real	CTCTTCA	CGATC
76	Des_111	Vila Real	CTAGGAC	CGATC
77	Dev_U02	Devon	ACGTA	CGATC
78	Dev_U03	Devon	AGAGT	CGATC
79	Dev_U04	Devon	ATGCT	CGATC
80	Dev_U05	Devon	GACTA	CGATC
81	Dev_U06	Devon	CAGTCAC	CGATC
82	Dev_U07	Devon	GCTAACA	CGATC
83	Dev_U08	Devon	ACACGAG	CGATC
84	Dev_U09	Devon	AGGACAC	CGATC
85	Dev_U10	Devon	TCAGA	CATCTGT
86	Dor_U11	Dorset	GATCG	CATCTGT
87	Dor_U12	Dorset	CATGA	CATCTGT
88	Dor_U13	Dorset	ATCGA	CATCTGT
89	Dor_U14	Dorset	TCGAG	CATCTGT
90	Dor_U15	Dorset	GTCAC	CATCTGT
91	Dor_U16	Dorset	GCATT	CATCTGT
92	Dor_U17	Dorset	CGATA	CATCTGT
93	Dor_U18	Dorset	TGCAACA	CATCTGT
94	Dor_U19	Dorset	CGTATCA	CATCTGT
95	Dor_U20	Dorset	CACAGAC	CATCTGT

MOLECULAR ECOLOGY
RESOURCES

Appendix 4 – Supplemental Figures

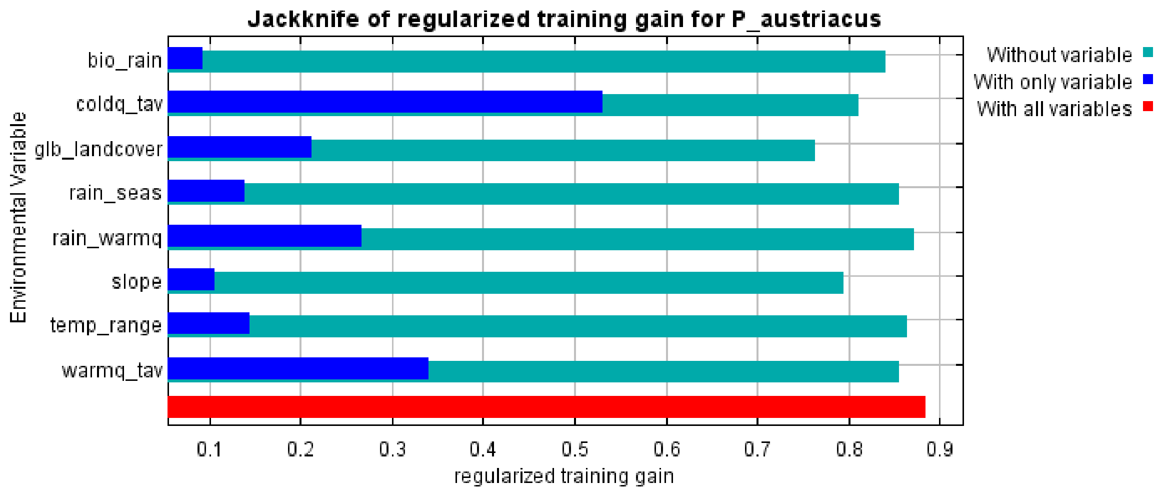

Figure S1 – Variables included in the ecological niche model and their relative contribution to the model in terms of their effect on increasing model gain when used in isolation (size of dark blue relative to red bar) and on decreasing model gain when omitted from the model (extent of reduction in light blue bars relative to the red bar). bio_rain = annual rainfall, coldq_tav = average temperature of the coldest quarter, glb_landcover = global land cover, rain_seas = rainfall seasonality, rain_warmq = summer rainfall, temp_range = annual temperature range (maximum minus minimum annual temperatures), warmq_tav = average temperature of the warmest quarter.

MOLECULAR ECOLOGY
RESOURCES

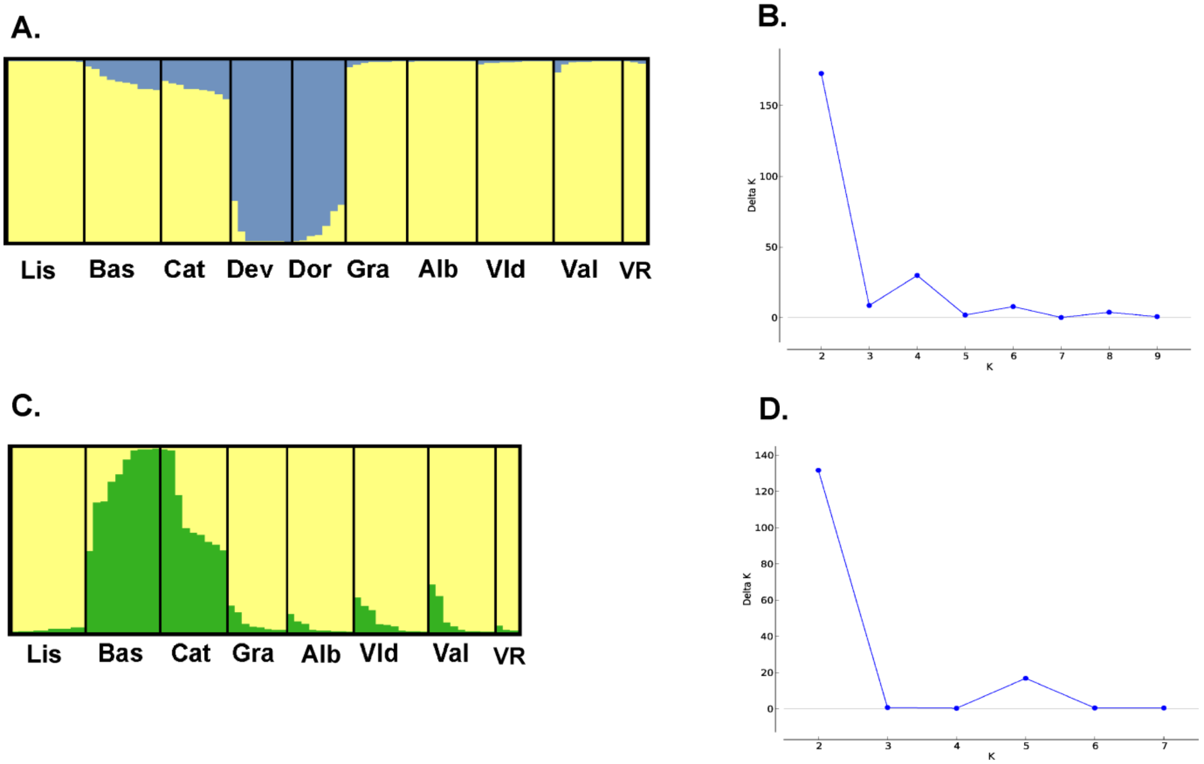

Figure S2 – Estimates of population structure based on individual-based assignment tests (STRUCTURE) and Evanno's method delta K (B, D) for the full *Plecotus austriacus* dataset (A, B) and within the Iberian cluster (C, D). Lis=Lisboa, Bas= Bizkaia, Cat=Girona, Dev=Devon, Dor=Dorset, Gra=Granada, Alb=Albacete, Vld=Valladolid, and Val=Valencia.

MOLECULAR ECOLOGY
RESOURCES

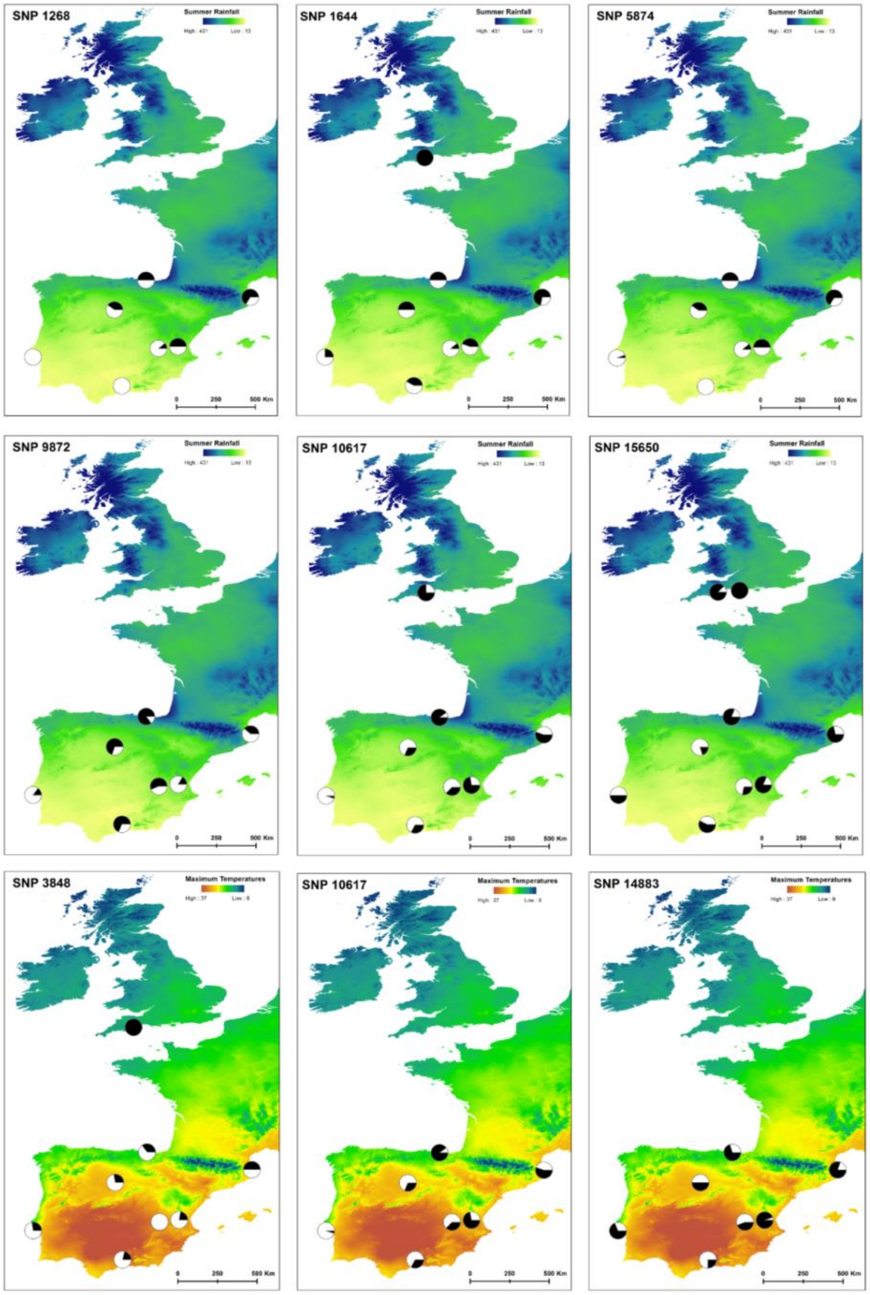

Figure S3 – Frequency of climate-adaptive SNPs in *Plecotus austriacus* populations, presented over climatic maps (summer rainfall and maximum temperatures). White represents alleles adapted to warmer and dryer conditions. Only Iberian genotypes are shown for SNPs that are only associated with climatic conditions in Iberia.

MOLECULAR ECOLOGY
RESOURCES

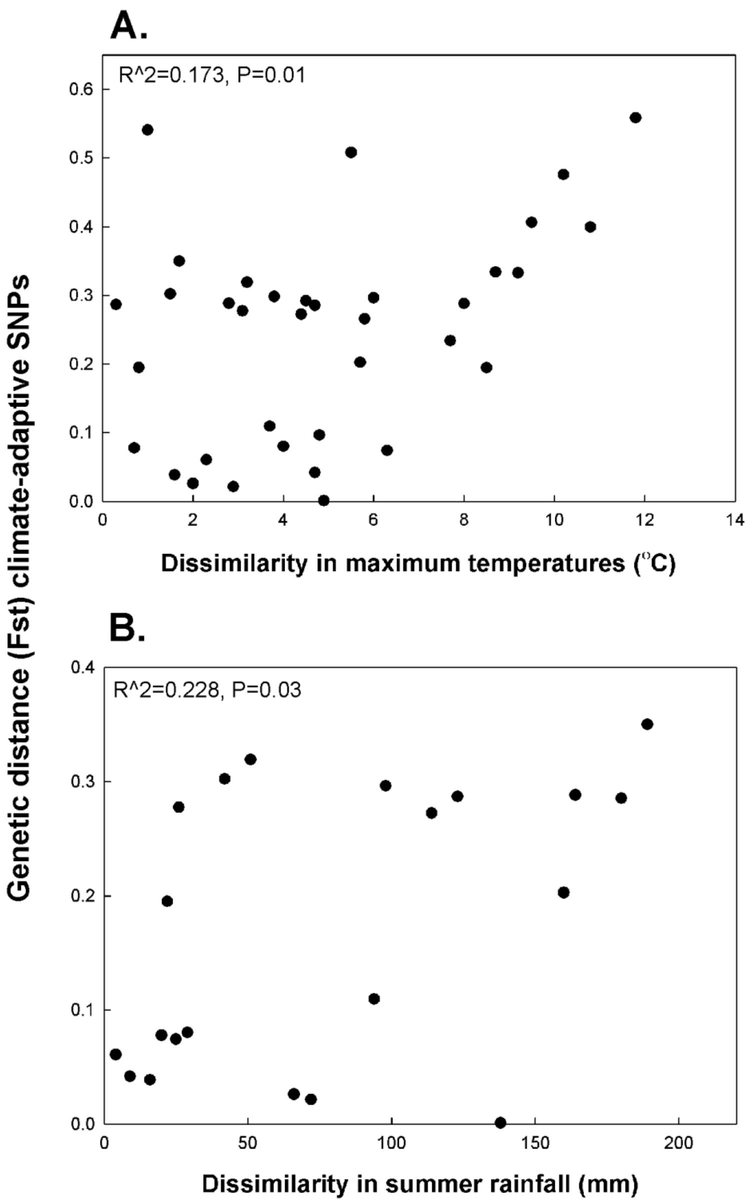

Figure S4 – Patterns of Isolation by Environment in *Plecotus austriacus* SNPs associated with climate-adaptive genetic variation across the whole study area (A) and within Iberia (B).
